# Label-free detection and size estimation of combustion-derived carbonaceous particles in a microfluidic approach†

**DOI:** 10.1039/d2na00262k

**Published:** 2022-06-21

**Authors:** Imran Aslam, Eduard Fron, Maarten B. J. Roeffaers

**Affiliations:** Centre for Membrane Separations, Adsorption, Catalysis, and Spectroscopy for Sustainable Solutions, Department of Microbial and Molecular Systems, KU Leuven Celestijnenlaan 200F 3001 Leuven Belgium maarten.roeffaers@kuleuven.be; Division of Molecular Imaging and Photonics, Department of Chemistry, KU Leuven Celestijnenlaan 200F 3001 Leuven Belgium

## Abstract

Detection and size estimation of combustion-derived carbonaceous particles (CDCPs) are important to understand their toxicity. Size determination of individual nano- and microparticles (NMPs) based on scattered light is a straightforward method. However, detection and sizing of CDCPs in biological samples based on scattering alone are not possible due to the compositional heterogeneity of NMPs present in biological samples. Label-free identification of CDCPs based on unique white light (WL) emission, using femtosecond (fs) pulsed near-infrared (NIR) lasers, has emerged as a reliable method even in complex biological samples. However, size estimation of CDCPs in biological samples using label-free techniques is still lacking. Here we report the development of a dual-channel multiphoton flow cytometry (DCMPFC) setup for label-free identification and size-determination of CDCPs in suspensions. Scattering intensity calibration with reference polystyrene (PS) nanoparticles (NPs) and Mie Theory allow us to determine the sizes of CDCPs in aqueous suspensions. Further, the relationship between particle sizes and WL emission intensity was determined, and the sizes of CDCPs in urine samples could also be estimated. This approach is believed to open new opportunities for the quantification and size determination of CDCPs, originating from exposure to air pollution, in liquid biopsies. This is an important step in determining the CDCP exposure of individual persons.

## Abstract 2

Combustion-derived carbonaceous particles constitute a major fraction of particulate matter air pollution. We estimated the sizes of the toxic CDCPs in aqueous suspensions based on scattered light by using reference polystyrene nanoparticles and Mie theory.

## Graphical abstract

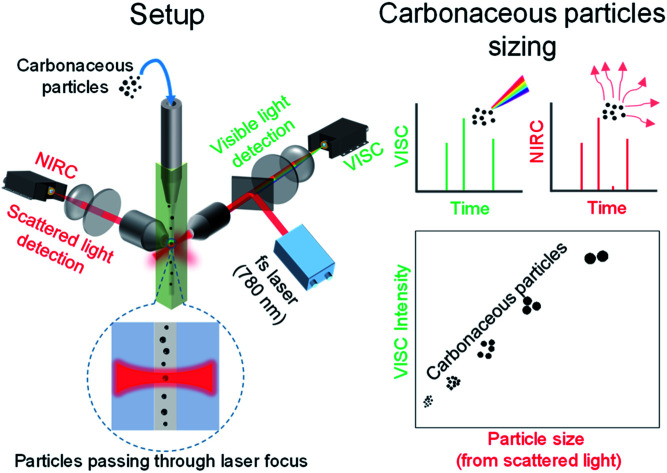

## Introduction

Combustion-derived carbonaceous particles (CDCPs) are omnipresent in the ambient environment,^1^ and they have various origins, with the main anthropogenic source being the incomplete combustion of fossil fuels and biomass burning.^2,3^ Their plentiful presence in the ambient atmosphere entails a large likelihood of their inhalation and subsequent translocation throughout the whole body.^4^ The inhalation and translocation are highly size-dependent with smaller CDCPs having been reported to be more dangerous as they can migrate more readily to different organs.^5–7^ As a consequence of increasing human exposure to CDCPs, monitoring the concentrations and sizes of CDCPs in different biological samples is essential for understanding their toxicological effects.

Different analytical approaches are used for the quantification and sizing of CDCPs in gaseous samples including laser-induced incandescence (LII), time-resolved LII, and the wide-angle light scattering technique.^8–10^ In addition, microscopy techniques such as transmission electron microscopy (TEM), scanning electron microscopy (SEM), and atomic force microscopy (AFM) are reliable methods for the size estimation of nanoparticles (NPs) in solid samples, but the obtained images may not be representative of the whole sample as a very small fraction of dried particles is observed.^11,12^ Furthermore, dynamic light scattering (DLS) and nanoparticle tracking analysis (NTA) are used for sizing NPs in suspensions based on light scattering and CDCPs’ Brownian motion.^13,14^ DLS and NTA are promising techniques for the size estimation of NPs in suspensions; however, these techniques cannot differentiate NPs in complex samples containing multiple types of scattering NP; further, a certain bias towards either higher or lower sizes exists depending on the polydispersity of the sample.^15,16^ Scattering properties of nanomaterials have also been used for other applications.^17,18^ Flow cytometry, using hydrodynamic focusing in a sheathed flow, is a widely used technique for high throughput and multiparameter analysis of nano-sized particles.^19–21^ Flow cytometry can provide information about the particle size, morphology, and biochemical attributes based on the scattered and fluorescence light from the particles passing through the laser focus.^22^ However, commercial flow cytometers have difficulties in measuring very small particles (<0.2 μm) and often rely on specific fluorescence labelling for particle identification.

Clearly, there is a need for the development of novel label-free analytical tools for the detection, quantification, and size estimation of CDCPs in complex media. Recently, we have reported that CDCPs can be detected in biological samples based on their unique white light (WL) emission under illumination with femtosecond (fs) pulsed near-infrared (NIR) lasers.^23,24^ In this work, we present the development of a dual-channel multiphoton flow cytometry (DCMPFC) setup, which combines light scattering with the unique WL emission from individual CDCPs passing through the focal spot of a fs-pulsed NIR laser for their label-free detection, quantification, and size estimation in a sheathed flow. The unique WL emission acts as a specific marker indicating the presence of CDCPs, whereas scattering detection can be used for their size estimation. Furthermore, a decreased background signal could be achieved through a very small probe volume and multiphoton excitation process resulting in the highly sensitive detection of individual CDCPs.^25,26^ In addition, using single-photon counting avalanche photodiodes (APDs) with excellent photon detection efficiency and limited aperture size (180 μm) further reduces the background enabling the highly sensitive detection of very small particles. The calibration of the setup was carried out using commercially available fluorescent polystyrene (fluo-PS) NPs. An experimental protocol was developed to study the aqueous suspensions prepared with 4 different types of commercially available CDCP. After calibrating the scattering signal of reference PS NP suspensions, the sizes of the unknown CDCPs could be determined using the Mie theory.^27^ Using this information, the dependence of WL emission intensity on the sizes of the CDCPs was estimated. To the best of our knowledge, this is the first report of the development of a multiphoton flow cytometry setup using two different channels for anti-Stokes and side-scatter detection. We believe that this system can find applications not only for the analysis of CDCPs but also other types of NP, as particle identification and sizing can be done using anti-Stokes and scattered light under fs-pulsed NIR laser illumination. Note that anti-Stokes emission can originate from multiple processes, *e.g.*, multi-photon excited fluorescence.^28^

## Results and discussion

### DCMPFC performance testing based on anti-Stokes and scattered light

**Fig. 1:**
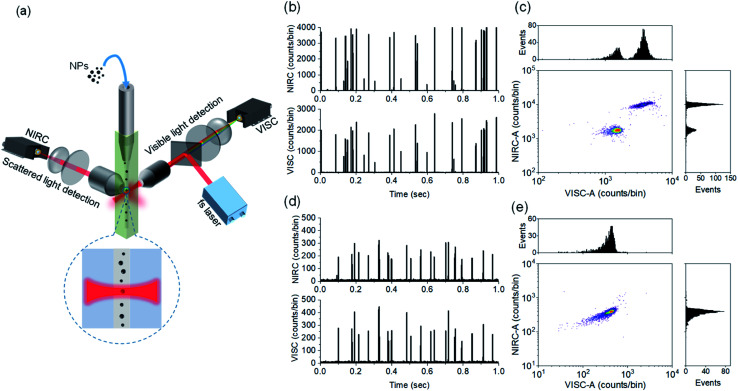
(a) Schematic of the setup design showing the analysis of individual NPs using a fs-pulsed NIR laser at 780 nm, where emitted anti-Stokes and scattered light from NPs are detected using VISC and NIRC, respectively. (b) Time traces of photon bursts from a mixture of 500 and 800 nm fluo-PS NPs passing through the laser focus. (c) Scatter plot with distribution histograms of VISC-A and NIRC-A showing separate population of 500 and 800 nm fluo-PS NPs. (d) Time traces of photon bursts from 200 nm fluo-PS NPs for VISC and NIRC seen above the background. (e) Scatter plot with distribution histograms for VISC-A and NIRC-A showing population of 200 nm fluo-PS NPs.

Our approach of dual-channel detection and size estimation of CDCPs in sheathed flow is inspired by the strategies of (1) fluorescence detection of single molecules in flow in combination with our finding that CDCPs under fs-pulsed NIR laser illumination emit WL and (2) sizing of macromolecules and nanoparticles based on scattering detection.^23,29–31^Fig. 1a (see Fig. S1† for detailed schematic illustration) shows the schematic of our in-house built DCMPFC setup. Hydrodynamic focusing of the sample stream into a very fine diameter (∼2.8 μm) flow ensures that each particle passes through the center of the focused laser beam (∼6.2 μm) generated by a perpendicularly placed 0.55 NA objective lens.

The alignment and calibration of the anti-Stokes (VISC) and scattering (NIRC) channels as well as the performance testing were carried out using fluo-PS NPs. These fluo-PS NPs are internally dyed with Fluoresbrite YG and provide sufficient fluorescence emission for VISC detection under two-photon excitation with a fs-pulsed NIR laser at 780 nm (Fig. S2†). First, we measured ultrapure water (filtered with a 0.22 μm filter) to serve as background and estimate any false positive signals due to scattering impurities (Fig. S3†). For ultrapure water, a few scattering events can be observed during the 100 s experiment (1 million data points of each 100 μs) in the NIRC. These events are very trivial (<0.5% in number) as compared to the events from reference fluo-PS NPs, and served as a background for measurements on NPs. Afterwards, we measured a mixture of 500 and 800 nm fluo-PS NPs as a reference.

Fig. 1b shows the VISC and NIRC time traces of photon bursts from a mixture of 500 and 800 nm fluo-PS NPs passing through the laser focus. In the VISC, the two-photon excited fluorescence from 500 and 800 nm fluo-PS NPs is clearly different with intensity peaks at around 800 and 2200 counts per bin, respectively. Also, the scattering intensity from both fluo-PS NPs is distinctly different with peaks at around 700 and 4000 counts per bin for 500 and 800 nm NPs, respectively. To avoid detector saturation, note that each bin is 100 μs, and the VISC and NIRC intensities were attenuated with ND filters (Table S1†). The photon bursts generated by individual particles passing through the laser focus show a good temporal correlation for VISC and NIRC (Fig. S4a†). Fig. 1c shows the scatter plot with correlated two-photon fluorescence (VISC) and scattering (NIRC) signals obtained from a mixture of 500 and 800 nm fluo-PS NPs; the plotted signals are obtained from the full peak burst area for VISC (VISC-A) and NIRC (NIRC-A) when an individual particle passes through the laser focus. Two different populations can be seen, linked to the two different NP sizes.

Next, similar measurements were performed on 200 nm fluo-PS NPs, now using a 0.8 OD ND filter in the NIRC channel. In Fig. 1d, the time traces of the fluorescence and scattering signal of individual 200 nm fluo-PS NPs passing through the laser focus can be seen. These signals are clearly above the background level. The peak heights for the VISC and NIRC reside around 260 and 250 counts per bin, respectively. The photon bursts generated from 200 nm fluo-PS NPs passing through the laser focus show a very strong temporal correlation between VISC and NIRC as about 96% peaks in both channels are time-correlated (Fig. S4b†). Fig. 1e shows the scatter plot with distribution histogram of the VISC-A and NIRC-A for 200 nm fluo-PS NPs. To determine the reproducibility of our measurements, the 200 nm fluo-PS NPs were repeatedly (5 times) measured. Based on these measurements, the relative standard deviations (RSDs), as the standard deviation divided by the mean, of 1.7% and 1.5% were achieved for the fluorescence and scattering detection, respectively, and for the number of fluo-PS NPs detected, the RSD is 4.3%. The signal-to-noise ratio (S/N), *i.e.*, the average signal of approx. 2200 fluo-PS NPs (200 nm) divided by the standard deviation of the background, is 108 and 51 for the VISC and NIRC, respectively.

Note that we refer to peak burst height for a visual distinction of different NPs in time traces, whereas the peak burst area is used for the scatter plot, histogram generation, and further quantitative data analysis as this value captures the total signal for each NP.

### Dual-channel detection of CDCPs based on WL emission and scattered light

**Fig. 2:**
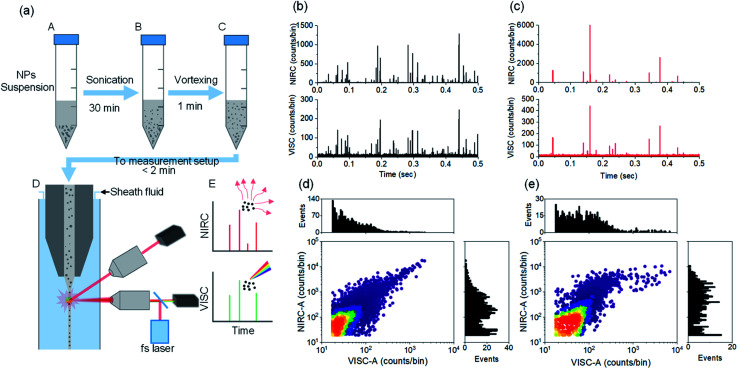
(a) Schematic of different steps used in the preparation of CDCP suspensions for measurements using the DCMPFC setup. The time between NP suspension preparation and the measurements is kept as short as possible to avoid aggregation. (b) Time traces of photon bursts from ufP90 NPs passing though the laser focus. (c) Time traces of photon bursts from fCB NPs passing through the laser focus. (d) Scatter plot with distribution histogram for VISC-A and NIRC-A of ufP90 showing the distribution of the population of ufP90. (e) Scatter plot with distribution histogram for VISC-A and NIRC-A of fCB showing the distribution of the population of fCB.

The label-free detection of CDCPs in complex samples can be performed based on unique WL emission under illumination with a fs-pulsed NIR laser using a multiphoton microscope. However, it is very time consuming to carry out the quantification and very challenging to perform the size determination of CDCPs in suspensions using a microscopy approach. Therefore, the DCMPFC setup employing single-particle analysis based on unique WL emission and concurrent scattering detection can provide an efficient solution. We have employed 4 different commercially available CDCPs with different sizes, according to their specifications, to test the abilities of the setup for their selective detection and size determination in aqueous suspensions using biologically relevant concentrations (20 μg mL^−1^).^32,33^ Before measuring using the DCMPFC setup, the non-incandescence related WL emission of the CDCP suspensions under illumination with a fs-pulsed NIR laser at 780 nm was double-checked (Fig. S2†). This emission spectrum shows that the unique WL emitted by CDCPs covers the whole visible spectrum and hence can be detected in the VISC. Fig. 2a shows a schematic diagram of different steps for sample preparation to perform measurements using the DCMPFC setup. To minimize aggregation, the time between sample preparation and measurement was kept as short as possible (<2 min). Fig. 2b, c, and S5a, b† show the emission and scattering from the 4 different CDCP suspensions as detected by the VISC and NIRC for (ufP90, fCB) and (ufPL, CCB), respectively. Time-correlated intensity peaks can be observed from the WL emission (VISC) and scattered light (NIRC) from the CDCPs in the case of all 4 different samples. In contrast to the rather uniform signal intensities recorded for monodisperse fluo-PS NPs, the signals of the CDCP suspensions show a varying intensity distribution which can be linked to the inherent size polydispersity of CDCPs. Further, ufP90 and ufPL have a larger fraction of low-intensity scattering and/or WL events compared to fCB and CCB. This is due to the ultrafine sizes of CDCPs in the case of ufP90 and ufPL. Fig. S7† shows a significant temporal correlation between VISC and NIRC for all 4 different CDCPs measured from the cross-correlation of VISC and NIRC. Based on the cross-correlation, about 80% of the peaks in the VISC and NIRC are time-correlated for ufP90 and ufPL, whereas about 50% and 40% peaks are time-correlated for fCB and CCB respectively. Fig. 2d and e show the scatter plots and the distribution histograms for VISC-A and NIRC-A for ufP90 and fCB, respectively. From the distribution histograms for VISC-A and NIRC-A, more events can be observed at higher counts in the case of fCB, which is due to the larger sizes of fCB particles (from manufacturer's data), as compared to ufP90 particles. Based on dual-channel detection, we could also quantify the number of particles detected per ml for fCB, CCB, ufP90, and ufPL in aqueous suspensions (Fig. S8†).

### Size determination of CDCPs and relationship between CDCP size and WL emission intensity

Due to the inherent size polydispersity of CDCPs resulting in varying intensity distribution in the VISC and NIRC, it is not possible to plot a calibration curve linking the emitted and/or scattered light intensity to the CDCP size for the reference samples. Therefore, an alternative approach is used to calculate the size of each CDCP from the light scattered when the particle is passing through the laser focus. By taking into account the optical configuration of our setup, Mie theory can be applied to calculate the CDCP particle sizes based on a calibration curve obtained for the reference PS NPs and taking the difference in the refractive index (RI) into consideration; the RI values (at 780 nm) used for PS and carbon are 1.579 + 0*i* and 1.950 + 0.8*i* respectively.^34–36^

**Fig. 3:**
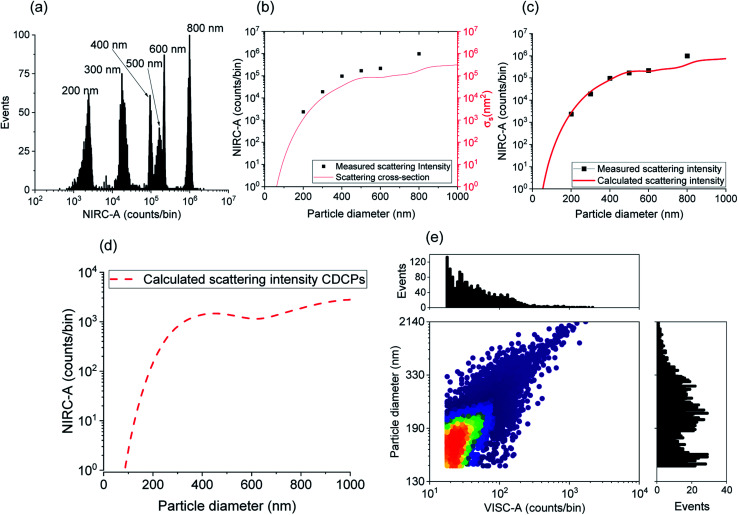
(a) Histogram of NIRC-A of PS NPs from 200 to 800 nm sizes used as a reference. (b) Measured scattering intensity (NIRC-A) plotted as a function of particle diameter. The mixture of 300, 400, and 600 nm PS NPs was measured separately. Comparison of the measured scattering intensity (from NIRC-A) and calculated scattering cross-section for different sizes of the reference PS NPs to determine the scaling factor. (c) Calculated scattering intensity is determined from the scattering cross-section using the scaling factor. The scaling factor is determined based on the median of the NIRC-A values obtained from 200 to 800 nm PS NPs. (d) Relevant scattering intensity for CDCPs (ufP90) obtained from the scattering cross-section. The calculation for the ND filter (OD 1.8) used for NIRC in the case of measurements on CDCPs was adjusted. (e) The particle diameter (of an approximated spherical particle) determined based on the calculation of the NIRC-A (counts per bin) from the scattering cross-section. It shows that the majority of CDCPs are around 200 to 330 nm in size. Particles with scattering intensity counts above 10^4^ are not included in this graph.

Fig. 3a shows the scattering intensity distribution histograms for NIRC-A obtained from 200 nm to 800 nm PS NPs; the average measured scattering intensity for each size is summarized in Fig. 3b. These experimental data were then fitted using the Mie theory (further details in the ESI†) to link the experimental scattering intensity to the calculated scattering cross-section *σ*_s_ using;^27^1
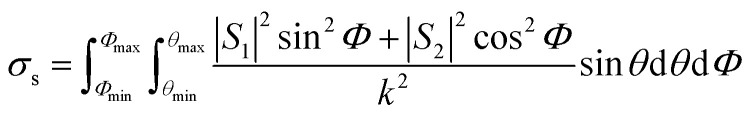
where *Φ* is the azimuthal angle with integration boundaries *Φ*_min_ and *Φ*_max_, *θ* is the polar angle with integration boundaries *θ*_min_ and *θ*_max_, *S*_1_ and *S*_2_ are the amplitude scattering matrix elements and 
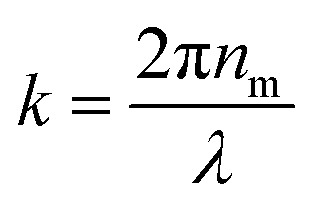
 is the wavenumber.

Fig. 3b shows the relationship between the sizes of the PS NPs and the measured scattering intensity, and the calculated scattering cross-section. The experimental scattering intensity from PS NPs is higher as compared to the calculated scattering cross-section. Hence, a setup characteristic scaling factor relating the measured scattering intensity to the calculated scattering cross-section can be determined using eqn (S5†) to obtain the calculated scattering intensity. Fig. 3c shows the measured scattering intensity and the scattering intensity calculated after applying the setup characteristic scaling factor (2.4) to the scattering cross-section for PS NPs. The same setup characteristic scaling factor is then applied to the scattering cross-section for the CDCPs of different sizes to obtain the relevant scattering intensities (Fig. 3d). The scattering intensity of CDCPs is higher than that of the PS NPs up to around 400 nm; thereafter, a small decrease in the scattering signal can be observed up to sizes of 600 nm. For sizes above 400 nm, the scattering intensity from CDCPs is lower than the scattering intensity from PS NPs, which is due to higher light absorption by CDCPs at longer wavelengths.^37^ Based on the calculated scattering intensity and the particle sizes for CDCPs (from Fig. 3d), we could estimate the sizes of the CDCPs measured in a sheathed flow using our setup. Fig. 3e shows the scatter plot and distribution histograms for VISC-A and the particle diameters estimated from the measured scattered intensities of NIRC-A for ufP90. Most of the particles are in the size range of about 200 nm (for ufP90) in aqueous suspension, which also corresponds to the hydrodynamic diameters measured using DLS (Table S2†) and NTA (Fig. S9†), and the sizes determined using SEM (Fig. S10†). The NTA data show that more than 90% of the particles are below 400 nm for all 4 different types of CDCP tested in this work. The sizes of all CDCPs determined using DLS are also below 400 nm except for CCB which is around 450 nm. This is because DLS is often biased towards larger particles as compared to NTA for polydisperse samples.^15,38^

**Fig. 4:**
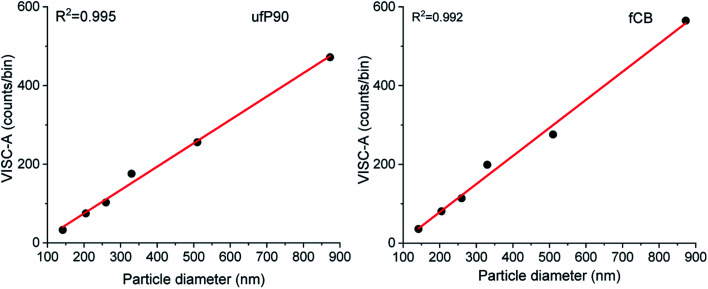
Estimation of the WL emission intensity as compared to CDCP size for ufP90 and fCB. A linear relationship between particle size and WL emission intensity was observed for CDCP sizes from 140 to 900 nm.

After size estimation of CDCPs based on the scattered light, the dependence of WL emission intensity on the sizes of CDCPs was determined (Fig. 4). For small particles from 140 nm to 900 nm, a linear relationship between CDCP size and WL emission intensity is observed. When the CDCP size increases above 1 μm, the relationship between CDCP size and WL emission intensity becomes non-linear (Fig. S11†). As CDCP sizes in biological samples are mostly within the small particle range, the linear relationship between CDCP size and WL emission intensity is more relevant for this study.^39^

### Applications in CDCP detection and size estimation in urine samples

**Fig. 5:**
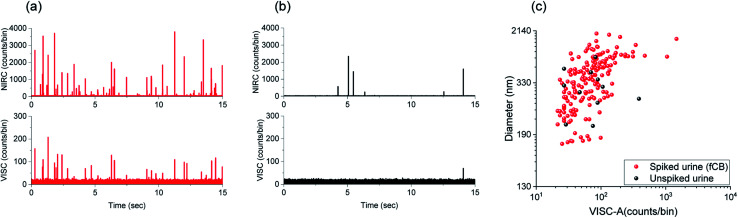
Measurements in the urine sample spiked with fCB and the unspiked urine sample. (a) Time traces from photon bursts in VISC and NIRC from the spiked urine sample. (b) Time traces of intensity peaks from the particles in the unspiked urine sample. (c) Dual-channel size determination of CDCPs detected in the spiked urine sample and unspiked urine sample.

The detection, quantification, and size estimation of CDCPs in urine samples can provide useful information about the exposure to CDCPs at the level of individual persons and their toxicological effects. Urine is a complicated medium containing multiple biological species, which might contribute to scattering, making detection and characterization of CDCPs in urine samples very challenging.^40–42^ We performed measurements in urine samples spiked with CDCPs and unspiked urine samples. The urine samples were treated by the addition of Tween 20 to lyse cells and solubilize proteins and lipids in urine to minimize the background scattering.^43^ Afterwards, the urine samples were vortexed and sonicated before measurements using our setup. Fig. 5a shows the intensity peaks in the VISC and NIRC from the urine samples spiked with 2 μg mL^−1^ fCB. More events are observed in the NIRC as compared to VISC. This indicates the presence of non-CDCP scattering species in the spiked urine samples, even after the addition of Tween 20, leading to intensity peaks in NIRC. However, about 50% of the NIRC peaks are correlated with VISC (see cross-correlation Fig. S13†). For the original urine samples, fewer events are detected, and about 15% of the NIRC events are correlated with VISC (Fig. 5b and S13†). Within the shown time trace of 15 s, 1 peak in the VISC was observed that also correlated with a peak in the NIRC; further, no VISC peaks are visible; however, 6 additional scattering peaks in the NIRC are visible, indicating the presence of other scattering particles in urine. As peaks from photon bursts in the VISC are only expected based on WL emission when a CDCP passes through the laser focus, this indicates the presence of CDCPs in urine samples of healthy individuals.^44^Fig. 5c shows the size estimation of particles detected in spiked urine samples and unspiked urine samples. The particle sizes determined for fCB in the spiked urine sample are within the same range as observed for fCB in MQ; about 65% of particles for fCB are 160 nm to 500 nm, whereas, larger sizes are also observed due to aggregation in urine. We could detect 11 particles with correlated signals in both channels in the unspiked urine within 3 min measurement time, and the sizes of the 10 detected particles range from 200 to 500 nm and that of 1 detected particle is about 900 nm. The detected particle sizes in urine samples are within the linear relationship range for CDCP sizes *versus* WL emission intensity that we determined earlier. Using a sample flow rate of 10 nl min^−1^, the average number of particles counted per ml (standard deviation) in the unspiked urine sample is 2.8 × 10^5^ (1.25 × 10^5^) (*n* = 3).^44^ The renal clearance of NPs has mostly been reported for sizes smaller than 10 nm in healthy individuals;^45,46^ therefore, detection of larger sizes using our technique indicate the agglomeration of CDCPs in the urine samples after renal clearance. We have tried to avoid potential external contamination of urine samples with CDCPs as much as possible from sample preparation to the measurements by using sterile vials and carrying out sample preparation in a laminar flow cabinet with air filtration.

## Conclusions

In conclusion, our work demonstrates for the first time that WL emission and scattering from CDCPs under illumination with a fs-pulsed NIR laser in combination with hydrodynamic focusing (using sheathed fluid) provides a practical approach for the label-free analysis of individual CDCPs. The setup was designed with two different channels to detect anti-Stokes and scattered light from the particles when illuminated using a fs-pulsed NIR laser at 780 nm. The background was reduced by focusing the sample stream to a very small diameter and using single-photon counting APDs with high photon detection efficiency. Reference PS NPs of different sizes were successfully detected and differentiated based on dual-channel detection using our setup. The dual-channel detection approach was then applied to the detection and quantification of CDCPs of different sizes in aqueous suspensions. By using Mie theory and reference PS NPs, we estimated the sizes of CDCPs in aqueous suspensions and determined the relationship between particle sizes and WL emission intensity. This approach was further applied to the detection, quantification, and size determination of CDCPs in urine samples. We believe that this setup could not only provide a unique solution to understand the toxicity of CDCPs in humans but also for quantification and size determination of various NMPs in suspensions for other applications.

## Experimental section

### DCMPFC setup

The DCMPFC system is designed to detect anti-Stokes and near-infrared scattered light simultaneously using a visible light channel (VISC) and near-infrared channel (NIRC), respectively (schematic of the setup in Fig. S1†). An ultrafast fiber laser (FemtoFErb 780, Toptica Photonics – Germany) with a central wavelength of 780 nm (<100 fs, 100 MHz) is used as an excitation source. A laser beam of 0.65 mm with an average laser power ∼35 mW at the objective was focused to a spot diameter of 6.2 μm (1/*e*^2^) unless otherwise stated. The incoming laser light and the anti-Stokes light from the CDCPs are collected by the same objective (G Plan Apo 50×, Mitutoyo – Japan). After collection by the objective, the anti-Stokes light is passed through a dichroic beam-splitter (FF750-SDi02, Semrock, Inc. USA) and filtered using a short-pass filter (ET750sp, Chroma – USA). Afterwards, the light was collected using an aspheric lens connected with a fiber coupler (PAF2P-18A – FiberPort, Thorlabs – Germany). The light is then guided through a multimode optical fiber (FG105LCA, Thorlabs – Germany) and detected by a single photon counting module with dark counts <1500 cps (SPCM-AQRH-10-FC-ND, Excelitas Technologies, Canada). The light scattered by the particles was collected with a 50× objective (LMPLFLN 50× objective, Olympus, Japan) at 90° to the incoming laser light and filtered using a bandpass filter (FB780-10 Thorlabs Inc., USA). Afterwards, the light is collected by an aspheric lens installed with a fiber coupler (PAF2P-18B – FiberPort, Thorlabs – Germany) and passed through a multimode optical fiber (FG105LCA, Thorlabs – Germany) before detection by a single photon counting module with dark counts <1500 cps (SPCM-AQRH-10-FC-ND, Excelitas Technologies, Canada). The fibers were shielded against any stray light to minimize background signals at the APDs. The anti-Stokes and scattered light were attenuated using neutral density (ND) filters to avoid saturation of the detectors (Table S1†).

### Data acquisition and processing

The signals from both APDs were collected using a data acquisition (DAQ) card (USB-6356, National Instruments – USA). A program in LabVIEW 2019 (National Instruments, USA) was written for data acquisition and photon counting using a DAQ card. The photon bursts from each particle passing through the laser focus were counted using the peak count feature in the LabVIEW. The detected photons were continuously counted in short time intervals (100 μs bin width) with a sampling rate of 10 kHz. The data processing is carried out using MATLAB ((R2020a, MathWorks, USA) as described by Habbersett *et al.*, and others.^47,48^ The data were processed using MATLAB to estimate actual photon count rates based on the dead time correction for APDs as described in the ESI (Fig. S14†). The peaks from photon bursts were counted above a certain threshold for the peak height and peak width. The threshold criteria for the peak height were set at a discriminator level of 3 to 7 standard deviations above the mean of the background signal. As we used 100 μs bin width as the data integration time, the criteria for the minimum and maximum peak widths were set at 100 μs and 400 μs, respectively. The peaks falling within the threshold criteria were further included in the data analysis. The burst areas from photon bursts were obtained based on the peak height and peak width. The background signal was estimated by measuring in blank ultrapure water. All data were acquired at room temperature. The graphs were plotted using Origin 2021b (OriginLab Corporation, USA).

### Fluidics system

All fluidics connections were purchased from Postnova Analytics GmbH, Germany, unless otherwise stated.

The sample stream was delivered through a 50 cm long quartz capillary having a 40 μm inner diameter and 240 μm outer diameter (New Objective, USA) with a tapered tip inserted inside a square-bored (250 × 250 μm^2^) quartz cuvette (Type 526 Flow Cytometer Cell, FireflySci, Inc., USA). The tapered tip of the capillary facilitates the smooth laminar flow of the sheath fluid.^49^ The sample volume was taken in a 1 mL plastic syringe (BD, Benelux), and delivered through a syringe pump (PHD 2000, Harvard Apparatus, USA) at a flow rate of 10 nl min^−1^ unless otherwise stated. The ultrapure water used as sheath fluid was delivered using a syringe pump (InfusionONE Syringe Pump, Darwin Microfluidics, France) after filtration with a 0.22 μm (Whatman Anotop, Sigma Aldrich, Belgium) syringe filter. The flow rate of the sheath fluid was 40 μl min^−1^ unless otherwise stated. Based on the dimensions of the cuvette and sheath flow rate, the average flow velocity in the center of the cuvette is calculated to be 21.33 mm s^−1^. The diameter of the sample stream was calculated to be ∼2.8 μm based on the sheath and sample flow rates using a simple model for hydrodynamic focusing.^25^ The particle transition time through the laser focus was calculated to be 288 μs based on the average flow velocity in the center of the cuvette and the laser spot diameter. Based on the overlap of the sample stream area and laser spot size, the calculated detection volume is approximately 38 femtolitres (fL). Based on Poisson's statistics, with a detection volume of 38 fL and a concentration of ∼1 × 10^8^ particles per mL, the probability for two particles to simultaneously pass through the laser focus is <0.1%.

### Polystyrene NPs and CDCP samples

For the calibration and performance evaluation of dual-channel detection, we used fluorescent (fluo) PS NPs of 200 nm, 500 nm, and 800 nm (Polysciences Europe GmbH, Germany). In addition, PS NPs of 300 nm, 400 nm, and 600 nm (Kisker Biotech, GmbH Germany) were also used for scattering detection as a reference. As a reference for CDCPs, we used four different types of commercially available carbon black (CB) NP which included ultrafine carbon black nanopowder (ufPL; PlasmaChem GmbH, Germany), ultrafine Printex 90 (ufP90; Orion Engineered Carbons, Germany), conductive carbon black nanopowder (CCB; US Research Nanomaterials, USA), and mesoporous carbon nanopowder (fCB; Sigma-Aldrich, Belgium). The mean aerodynamic diameters from the manufacturer are 13, 14, 150, and <500 nm for ufPL, ufP90, CCB, and fCB, respectively.

### Sample preparation for DCMPFC

The fluo-PS NPs (200 nm, 500 nm, and 800 nm) for the calibration and performance analysis of dual-channel detection were used at concentration ∼1 × 10^8^ particles per mL. Other PS NPs (300 nm, 400 nm, and 600 nm) for scattering detection were used at concentrations 1–5 × 10^8^ particles per mL in ultrapure water (MilliQ; Merck Millipore, Belgium). The stock suspension of CDCPs was prepared in ultrapure water with 0.1% Tween 20 at 2 mg mL^−1^ concentration. This stock suspension was stored at 4 °C in the dark until further use. Before measurements, the stock suspension was sonicated for 30 minutes at 40 kHz and then diluted to the desired concentrations of 20 μg mL^−1^ or 2 μg mL^−1^. Afterwards, the diluted suspension is vortexed for 1 minute and sonicated for 30 minutes, and measurements were performed immediately. For measurements on urine samples, the urine samples were collected from seemingly healthy volunteers and stored in conical vials at −18 °C for future use. For measurements in unspiked and spiked urine samples, 0.1–0.5% Tween 20 is added in the urine to lyse cells and solubilize proteins and lipids which can result in unwanted signals at the scattering channel. Afterwards, the urine sample was sonicated for 10 minutes at 40 kHz and vortexed for 30 seconds. The measurements were performed immediately (∼2 minutes) after sample preparation to avoid aggregation of the particles. The urine samples were spiked with 2 μg mL^−1^ fCB. All experiments were performed in accordance with the guidelines laid down in the EU regulation 2016/679, the Belgian Law on patients' rights of 22/08/2002, and the institutional guidelines. The protocol was approved by the Ethics Committee Research UZ/KU Leuven (Application#: S63662). Informed consent was obtained from human participants of this study.

## Conflicts of interest

The authors declare no conflicts of interest.

## Supplementary Material

NA-004-D2NA00262K-s001

## References

[cit1] Donaldson K., Tran L., Jimenez L. A., Duffin R., Newby D. E., Mills N., MacNee W., Stone V. (2005). Combustion-Derived Nanoparticles: A Review of Their Toxicology Following Inhalation Exposure. Part. Fibre Toxicol..

[cit2] Gieré R., Querol X. (2010). Solid Particulate Matter in the Atmosphere. Elements.

[cit3] Reşitoğlu I. A., Altinişik K., Keskin A. (2015). The Pollutant Emissions from Diesel-Engine Vehicles and Exhaust Aftertreatment Systems. Clean Technol. Environ. Policy.

[cit4] Mills N. L., Amin N., Robinson S. D., Anand A., Davies J., Patel D., De La Fuente J. M., Cassee F. R., Boon N. A., MacNee W., Millar A. M., Donaldson K., Newby D. E. (2006). Do Inhaled Carbon Nanoparticles Translocate Directly into the Circulation in Humans?. Am. J. Respir. Crit. Care Med..

[cit5] Terzano C., Di Stefano F., Conti V., Graziani E., Petroianni A. (2010). Air Pollution Ultrafine Particles: Toxicity beyond the Lung. Eur. Rev. Med. Pharmacol. Sci..

[cit6] Donaldson K., Stone V., Clouter A., Renwick L., MacNee W. (2001). Ultrafine Particles. J. Occup. Environ. Med..

[cit7] Cesaroni G., Forastiere F., Stafoggia M., Andersen Z. J., Badaloni C., Beelen R., Caracciolo B., De Faire U., Erbel R., Eriksen K. T., Fratiglioni L., Galassi C., Hampel R., Heier M., Hennig F., Hilding A., Hoffmann B., Houthuijs D., Jöckel K. H., Korek M., Lanki T., Leander K., Magnusson P. K. E., Migliore E., Ostenson C. G., Overvad K., Pedersen N. L., Pekkanen J. J., Penell J., Pershagen G., Pyko A., Raaschou-Nielsen O., Ranzi A., Ricceri F., Sacerdote C., Salomaa V., Swart W., Turunen A. W., Vineis P., Weinmayr G., Wolf K., De Hoogh K., Hoek G., Brunekreef B., Peters A. (2014). Long Term Exposure to Ambient Air Pollution and Incidence of Acute Coronary Events: Prospective Cohort Study and Meta-Analysis in 11 European Cohorts from the Escape Project. BMJ.

[cit8] Schulz C., Kock B. F., Hofmann M., Michelsen H., Will S., Bougie B., Suntz R., Smallwood G. (2006). Laser-Induced Incandescence: Recent Trends and Current Questions. Appl. Phys. B: Lasers Opt..

[cit9] Sun Z. W., Gu D. H., Nathan G. J., Alwahabi Z. T., Dally B. B. (2015). Single-Shot, Time-Resolved Planar Laser-Induced Incandescence (TiRe-LII) for Soot Primary Particle Sizing in Flames. Proc. Combust. Inst..

[cit10] Oltmann H., Reimann J., Will S. (2012). Single-Shot Measurement of Soot Aggregate Sizes by Wide-Angle Light Scattering (WALS). Appl. Phys. B: Lasers Opt..

[cit11] Fissan H., Ristig S., Kaminski H., Asbach C., Epple M. (2014). Comparison of Different Characterization Methods for Nanoparticle Dispersions before and after Aerosolization. Anal. Methods.

[cit12] Liu F. K., Chang Y. C., Ko F. H., Chu T. C., Dai B. T. (2003). Rapid Fabrication of High Quality Self-Assembled Nanometer Gold Particles by Spin Coating Method. Microelectron. Eng..

[cit13] Elizalde O., Leal G. P., Leiza J. R. (2000). Particle Size Distribution Measurements of Polymeric Dispersions: A Comparative Study. Part. Part. Syst. Charact..

[cit14] Malloy A., Carr B. (2006). Nanoparticle Tracking Analysis - The HaloTM System. Part. Part. Syst. Charact..

[cit15] Filipe V., Hawe A., Jiskoot W. (2010). Critical Evaluation of Nanoparticle Tracking Analysis (NTA) by NanoSight for the Measurement of Nanoparticles and Protein Aggregates. Pharm. Res..

[cit16] Dragovic R. A., Gardiner C., Brooks A. S., Tannetta D. S., Ferguson D. J. P., Hole P., Carr B., Redman C. W. G., Harris A. L., Dobson P. J., Harrison P., Sargent I. L. (2011). Sizing and Phenotyping of Cellular Vesicles Using Nanoparticle Tracking Analysis. Nanomed. Nanotechnol. Biol. Med..

[cit17] Bhaskar S., Das P., Moronshing M., Rai A., Subramaniam C., Bhaktha S. B. N., Ramamurthy S. S. (2021). Photoplasmonic Assembly of Dielectric-Metal, Nd2O3-Gold Soret Nanointerfaces for Dequenching the Luminophore Emission. Nanophotonics.

[cit18] Bhaskar S., Singh A. K., Das P., Jana P., Kanvah S., Bhaktha B. N. S., Ramamurthy S. S. (2020). Superior Resonant Nanocavities Engineering on the Photonic Crystal-Coupled Emission Platform for the Detection of Femtomolar Iodide and Zeptomolar Cortisol. ACS Appl. Mater. Interfaces.

[cit19] Shapiro H. M. , Learning Flow Cytometry, in Practical Flow Cytometry, 2005, 10.1002/0471722731.ch2

[cit20] Steen H. B. (2004). Flow Cytometer for Measurement of the Light Scattering of Viral and Other Submicroscopic Particles. Cytometry.

[cit21] Ibuki Y., Toyooka T. (2012). Nanoparticle Uptake Measured by Flow Cytometry. Methods Mol. Biol..

[cit22] Macey M. G. , Flow Cytometry: Principles and Applications, 2007, 10.1007/978-1-59745-451-3

[cit23] Bové H., Steuwe C., Fron E., Slenders E., D’Haen J., Fujita Y., Uji-I H., Vandeven M., Roeffaers M., Ameloot M. (2016). Biocompatible Label-Free Detection of Carbon Black Particles by Femtosecond Pulsed Laser Microscopy. Nano Lett..

[cit24] Aslam I., Roeffaers M. B. J. (2021). Unique Emissive Behavior of Combustion-Derived Particles under Illumination with Femtosecond Pulsed near-Infrared Laser Light. Nanoscale Adv..

[cit25] Zarrin F., Dovichi N. J. (1985). Sub-Picoliter Detection with the Sheath Flow Cuvette. Anal. Chem..

[cit26] Rubart M. (2004). Two-Photon Microscopy of Cells and Tissue. Circ. Res..

[cit27] de Rond L., Coumans F. A. W., Nieuwland R., van Leeuwen T. G., van der Pol E. (2018). Deriving Extracellular Vesicle Size From Scatter Intensities Measured by Flow Cytometry. Curr. Protoc. Cytom..

[cit28] Maestro L. M., Rodriguez E. M., Vetrone F., Naccache R., Ramirez H. L., Jaque D., Capobianco J. A., Solé J. G. (2010). Nanoparticles for
Highly Efficient Multiphoton Fluorescence Bioimaging. Opt. Express.

[cit29] Keller R. A., Ambrose W. P., Goodwin P. M., Jett J. H., Martin J. C., Wu M. (1996). Single-Molecule Fluorescence Analysis in Solution. Appl. Spectrosc..

[cit30] Tian Y., Ma L., Gong M., Su G., Zhu S., Zhang W., Wang S., Li Z., Chen C., Li L., Wu L., Yan X. (2018). Protein Profiling and Sizing of Extracellular Vesicles from Colorectal Cancer Patients via Flow Cytometry. ACS Nano.

[cit31] Zhang W., Tian Y., Hu X., He S., Niu Q., Chen C., Zhu S., Yan X. (2018). Light-Scattering Sizing of Single Submicron Particles by High-Sensitivity Flow Cytometry. Anal. Chem..

[cit32] Li N., Hao M., Phalen R. F., Hinds W. C., Nel A. E. (2003). Particulate Air Pollutants and Asthma: A Paradigm for the Role of Oxidative Stress in PM-Induced Adverse Health Effects. Clin. Immunol..

[cit33] Bové H., Devoght J., Rasking L., Peters M., Slenders E., Roeffaers M., Jorge-Peñas A., Van Oosterwyck H., Ameloot M. (2018). Combustion-Derived Particles Inhibit in Vitro Human Lung Fibroblast-Mediated Matrix Remodeling. J. Nanobiotechnol..

[cit34] Bohren C. F. , Absorption and Scattering of Light by Small Particles*,* 1983, 10.1088/0031-9112/35/3/025

[cit35] Sultanova N. G., Kasarova S. N., Nikolov I. D. (2013). Characterization of Optical Properties of Optical Polymers. Opt. Quantum Electron..

[cit36] Hagemann H.J. , Gudat W. and Kunz C., DESY Report SR-74-7, 1974

[cit37] Bond T. C., Bergstrom R. W. (2006). Light Absorption by Carbonaceous Particles: An Investigative Review. Aerosol Sci. Technol..

[cit38] Stetefeld J., McKenna S. A., Patel T. R. (2016). Dynamic Light Scattering: A Practical Guide and Applications in Biomedical Sciences. Biophys. Rev..

[cit39] Kittelson D. B., Watts W. F., Johnson J. P., Remerowki M. L., Ische E. E., Oberdörster G., Gelein R. M., Elder A., Hopke P. K., Kim E., Zhao W., Zhou L., Jeong C. H. (2004). On-Road Exposure to Highway Aerosols. 1. Aerosol and Gas Measurements. Inhalation Toxicol..

[cit40] Ouma J., Septien S., Velkushanova K., Pocock J., Buckley C. (2016). Characterization of Ultrafiltration of Undiluted and Diluted Stored Urine. Water Sci. Technol..

[cit41] Putnam D. F. , Composition and Concentrative Properties of Human Urine, in NASA Contractor Report: NASA CR-1802, 1971

[cit42] Lou Y., He W., Song Z. (2020). Aggregation of Nanochemical Microcrystals in Urine Promotes the Formation of Urinary Calculi. J. Chem..

[cit43] Islam M. S., Aryasomayajula A., Selvaganapathy P. R. (2017). A Review on Macroscale and Microscale Cell Lysis Methods. Micromachines.

[cit44] Saenen N. D., Bové H., Steuwe C., Roeffaers M. B. J., Provost E. B., Lefebvre W., Vanpoucke C., Ameloot M., Nawrot T. S. (2017). Children’s Urinary Environmental Carbon Load: A Novel Marker Reflecting Residential Ambient Air Pollution Exposure?. Am. J. Respir. Crit. Care Med..

[cit45] Choi C. H. J., Zuckerman J. E., Webster P., Davis M. E. (2011). Targeting Kidney Mesangium by Nanoparticles of Defined Size. Proc. Natl. Acad. Sci. U. S. A..

[cit46] Du B., Yu M., Zheng J. (2018). Transport and Interactions of Nanoparticles in the Kidneys. Nat. Rev. Mater..

[cit47] Habbersett R. C., Jett J. H. (2004). An Analytical System Based on a Compact Flow Cytometer for DNA Fragment Sizing and Single-Molecule Detection. Cytometry.

[cit48] Yang L., Zhu S., Hang W., Wu L., Yan X. (2009). Development of an Ultrasensitive Dual-Channel Flow Cytometer for the Individual Analysis of Nanosized Particles and Biomolecules. Anal. Chem..

[cit49] Van Orden A., Cai H., Goodwin P. M., Keller R. A. (1999). Efficient Detection of Single DNA Fragments in Flowing Sample Streams by Two-Photon Fluorescence Excitation. Anal. Chem..

