# Supplementary material for: Label-free detection and size estimation of combustion-derived carbonaceous particles in a microfluidic approach
Source: Nanoscale Adv. 2022 Jun 21;4(15):3272–81. doi: 10.1039/d2na00262k (PMC9419628; doi:10.1039/d2na00262k)
Supplement: NA-004-D2NA00262K-s001 [file NA-004-D2NA00262K-s001.pdf]

Electronic Supplementary Material (ESI) for Nanoscale Advances.
This journal is © The Royal Society of Chemistry 2022

Supporting Information

Label-Free Detection and Size Estimation of Combustion-Derived Carbonaceous Particles in a Microfluidic Approach

Imran Aslam,[a] *Eduard Fron,*[b] *and Maarten B.J. Roeffaers* [a] *

[a] Centre for Membrane Separations, Adsorption, Catalysis, and Spectroscopy for Sustainable Solutions, Department of Microbial and Molecular Systems, KU Leuven Celestijnenlaan 200F, 3001 Leuven, Belgium

[b] Division of Molecular Imaging and Photonics, Department of Chemistry, KU Leuven, Celestijnenlaan 200F, 3001 Leuven, Belgium

*Corresponding author:

Email: maarten.roeffaers@kuleuven.be

Experimental section

Emission spectra measurements

The emission spectra were measured on 2 mg/mL fCB suspension and, fluorescent polystyrene (fluo-PS) NPs inside Ibidi µ-Slide 8 well plates (Ibidi GmbH, Germany). The emission spectra were measured using a commercial microscope (DMi8, Leica Microsystems GmbH, Germany) equipped with tunable NDD detectors in a commercial system (Leica SP8 Dive, Leica Microsystems GmbH, Germany). The 780 nm light from a tunable laser (MaiTai Insight X3, 80 MHz, 120 fs, SpectraPhysics, USA) was focused on the samples using a water immersion objective (HC PL IRAPO 40x, Leica Microsystems GmbH, Germany). The light emitted by the particles is collected by the same objective and separated from the laser light using an Acousto-Optical Beam Splitter (AOBS, Leica Microsystems CMS GmbH, Germany). Afterwards, the emitted light is collected by a 4Tune non-descanned detection unit in the microscope; the 4Tune detection unit allows spectral detection using non-descanned detectors (Leica HyD SMD, Leica Microsystems GmbH, Germany). The light emitted by the particles was detected over the whole visible region from 380 nm to 640 nm. All data were collected using the Leica Application Suite X software (Leica Microsystems GmbH, Germany). The data were processed using Origin 2021b (OriginLab Corporation, USA).

Dynamic light scattering (DLS) characterization

Hydrodynamic diameters of the particles suspended in ultrapure water were measured by dynamic light scattering (DLS) (Brookhaven Instruments Corp., USA). A standard disposable cuvette was used for nanoparticle suspensions to perform the analysis. The measurement data were collected for 2 minutes with multiple measurements on each sample to improve the accuracy.

Nanoparticle tracking analysis (NTA)

The hydrodynamic diameters of particles suspended in ultrapure water were also measured using nanoparticles tracking analysis (NTA) (NanoSight, UK). The measurements were performed on the same sample multiple times for repeatability.

Scanning electron microscopy (SEM) characterization

The dried nanoparticles were observed using a scanning electron microscope (FEI Quanta 250 FEG) with an acceleration voltage of 20 kV.

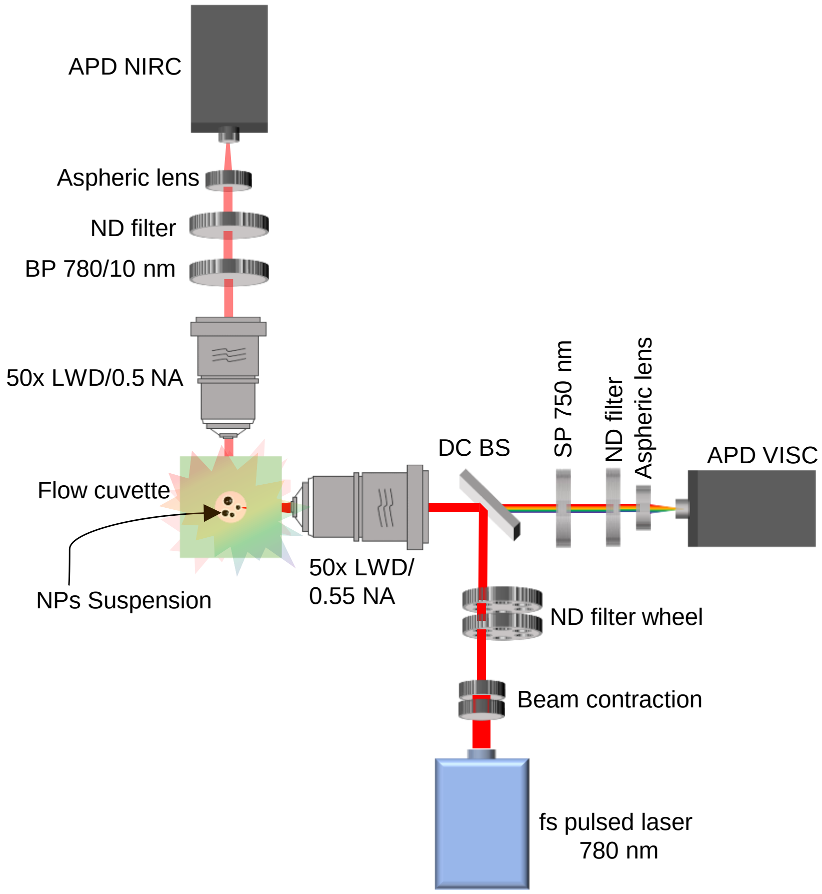

Figure S1: Schematic of the dual-channel multiphoton flow cytometry (DCMPFC) setup.

fs; femtosecond, DC; dichroic, BS; beam splitter, SP; short pass, ND; neutral density , BP; bandpass, APD; avalanche photodiode, LWD; long working distance

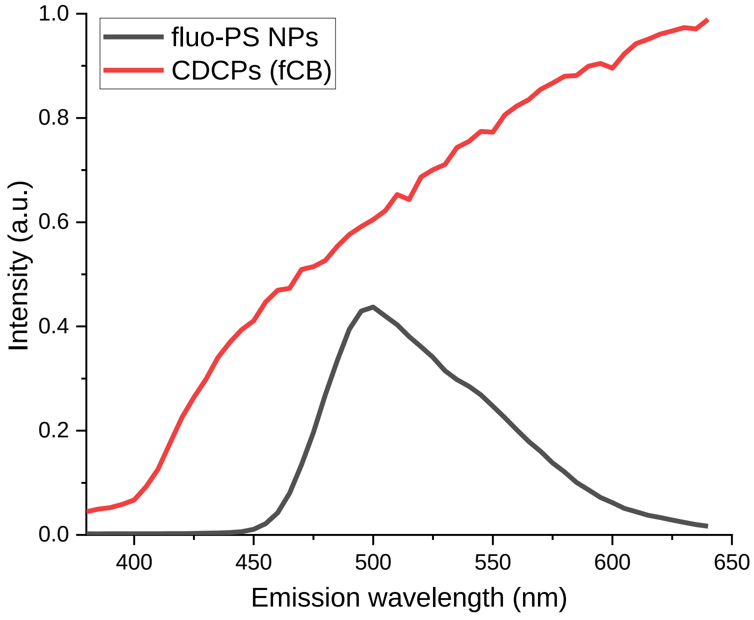

Figure S2: Emission spectra of 500 nm fluo-PS NPs and CDCPs under illumination with a fs-pulsed NIR laser at 780 nm. The laser power is adjusted to get enough emission intensity for both samples.

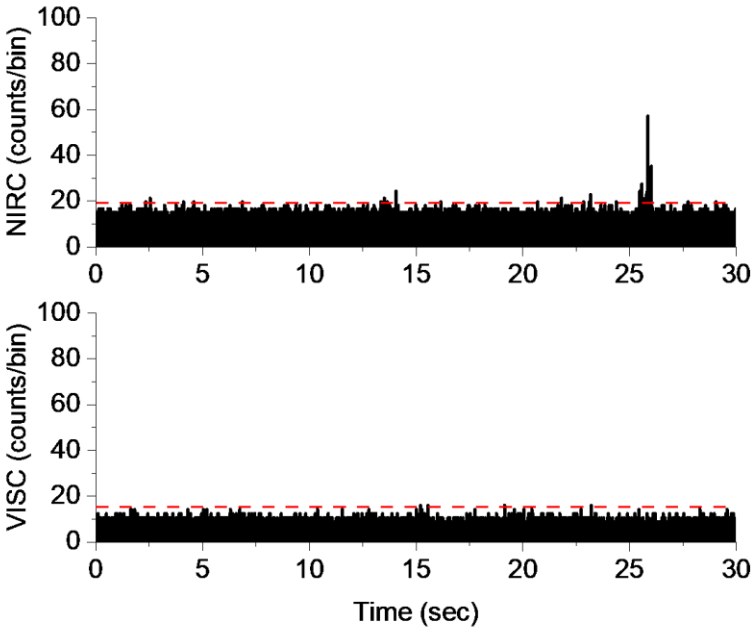

Figure S3: Time traces of photon bursts from blank MQ. No significant peaks can be observed in VISC, whereas a few peaks can be observed in NIRC within 30 sec. measurement time indicating the presence of the impurities in blank MQ. The red dashed line shows the threshold settings for background removal.

(a)

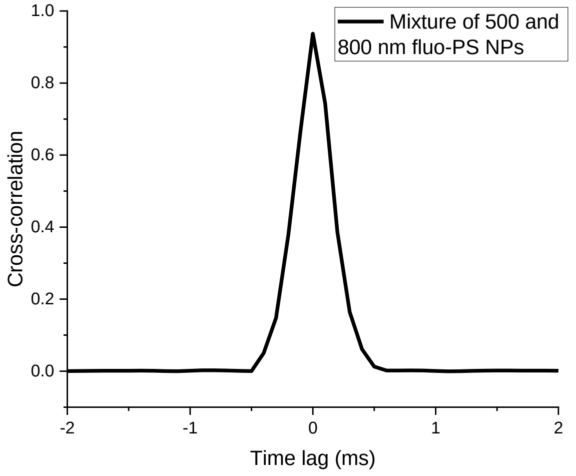

(b)

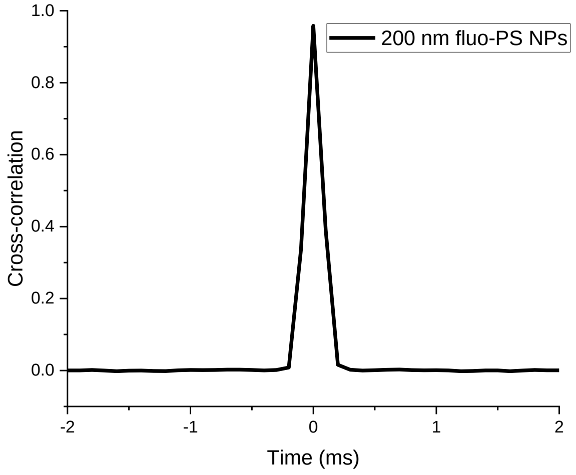

Figure S4: (a) Cross- correlation between NIRC and VISC from a mixture of 500nm, and 800 nm fluo-PS NPs. (b) Cross-correlation between VISC and NIRC for 200 nm fluo-PS NPs. Cross-correlation is measured using a MATLAB routine. The cross-correlation function measures the similarity between VISC and lagged versions of NIRC time traces as a function of the lag.

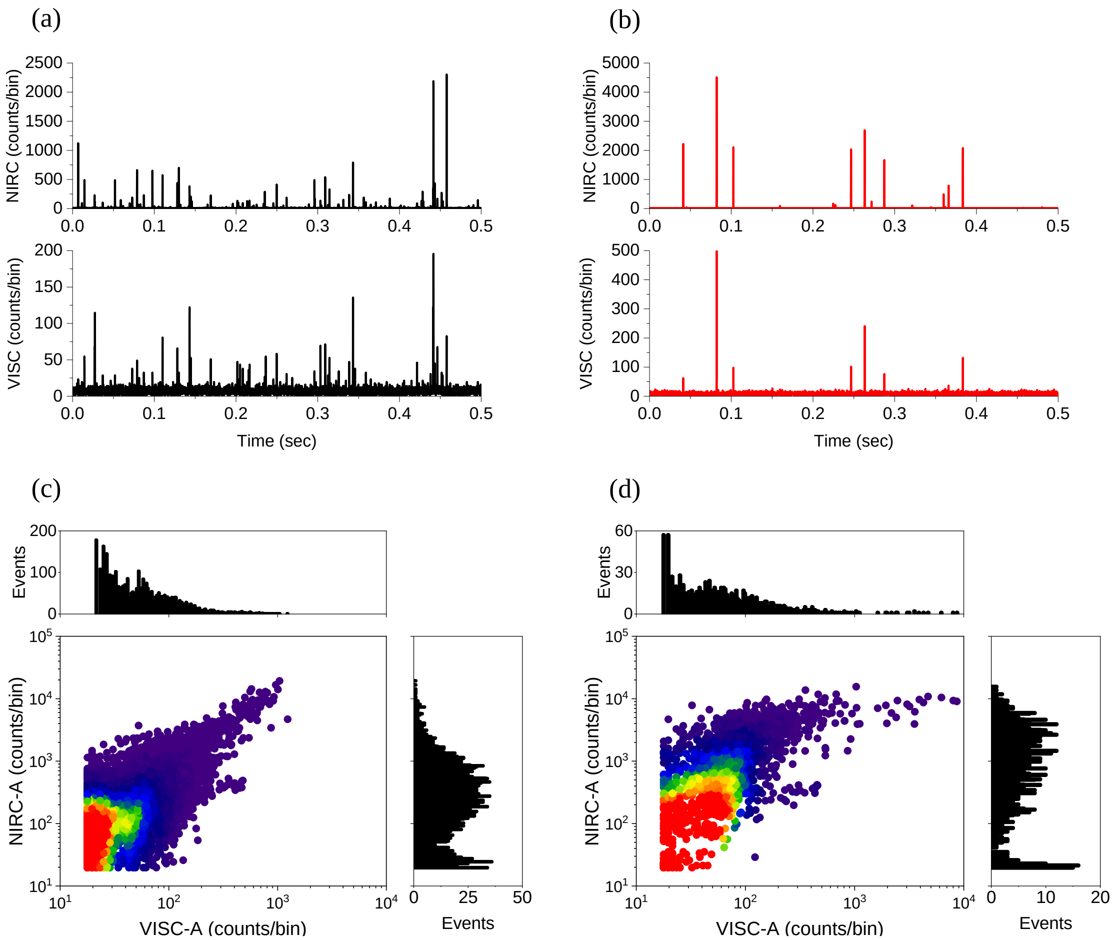

Figure S5: Dual-channel measurements on ufPL and CCB. (a) Time traces of photon bursts from ufPL NPs passing through the laser focus. (b) Time traces of photon bursts from CCB NPs passing through the laser focus. (c) Scatter plot with histograms for VISC-A and NIRC-A of ufPL. (d) Scatter plot with histograms for VISC-A and NIRC-A of CCB showing the distribution of the population of CCB.

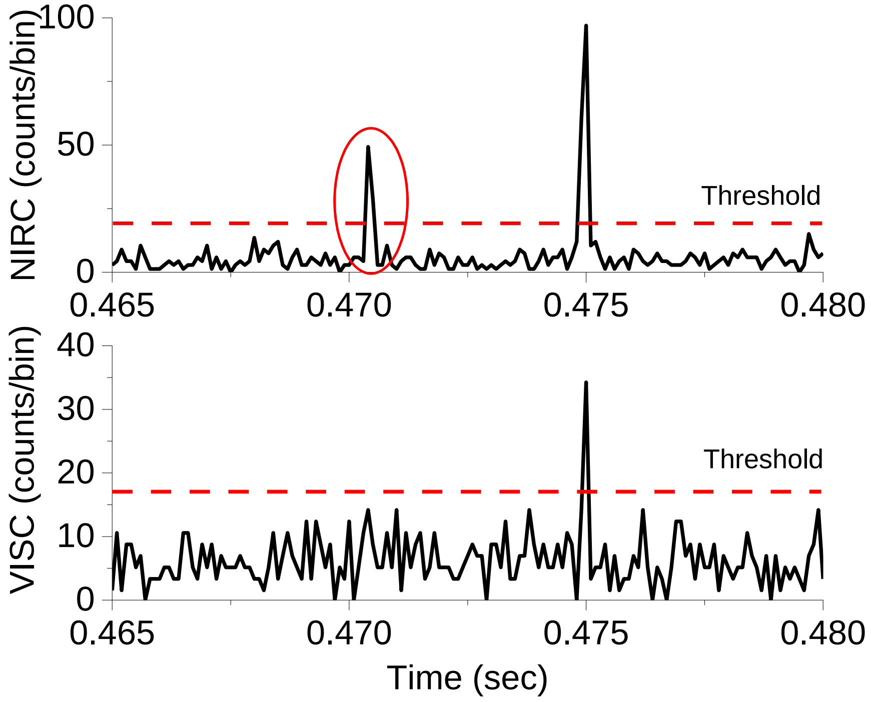

Figure S6: For very small CDCPs, scattering peaks can be seen slightly above the background in NIRC, but no peaks visible in VISC which could be due to the very low WL emission intensity. The binwidth is 100 μs. The threshold is is set at mean+5SD from measurements on blank MQ.

SD: standard deviation

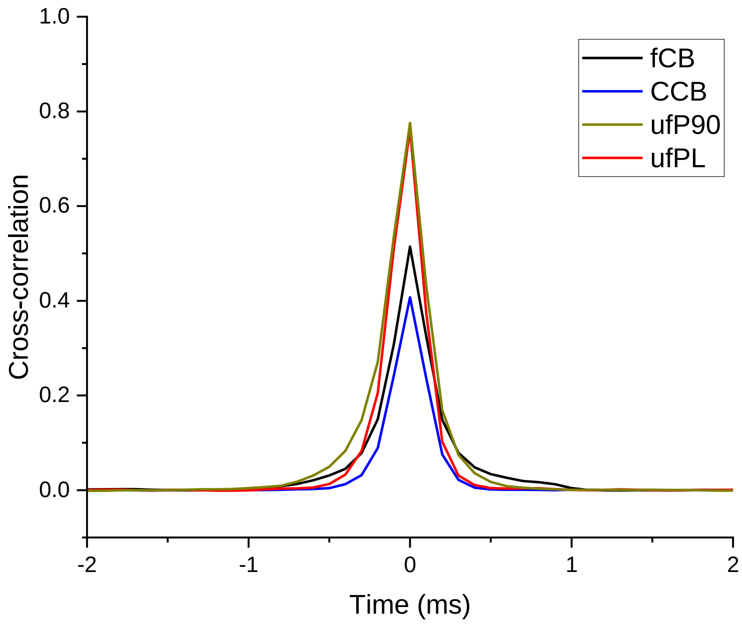

Figure S7: Cross-correlation between VISC and NIRC shows strong peaks a t=0 time lag for all 4 CDCPs used in this study.

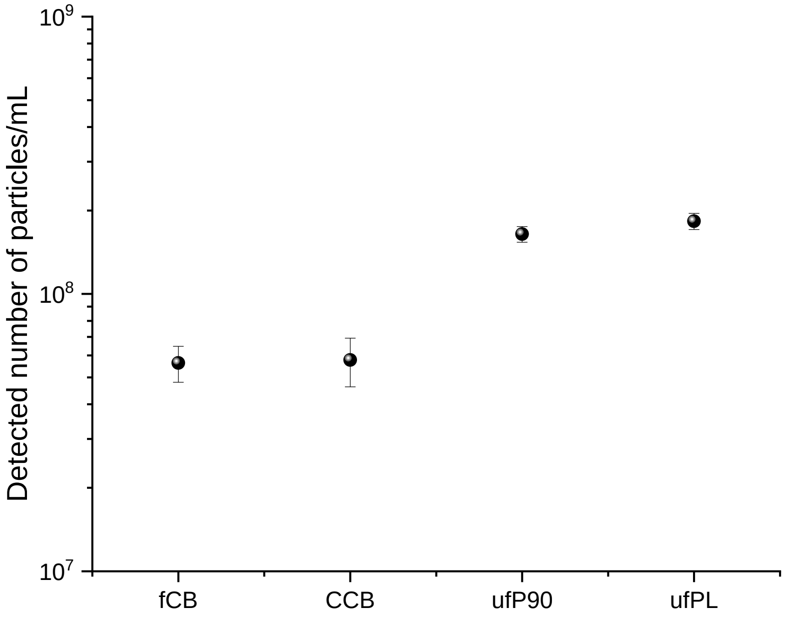

Figure S8: Number of particles counted per mL based on dual-channel detection using DCMPFC. The data are average ±SD (n=5).

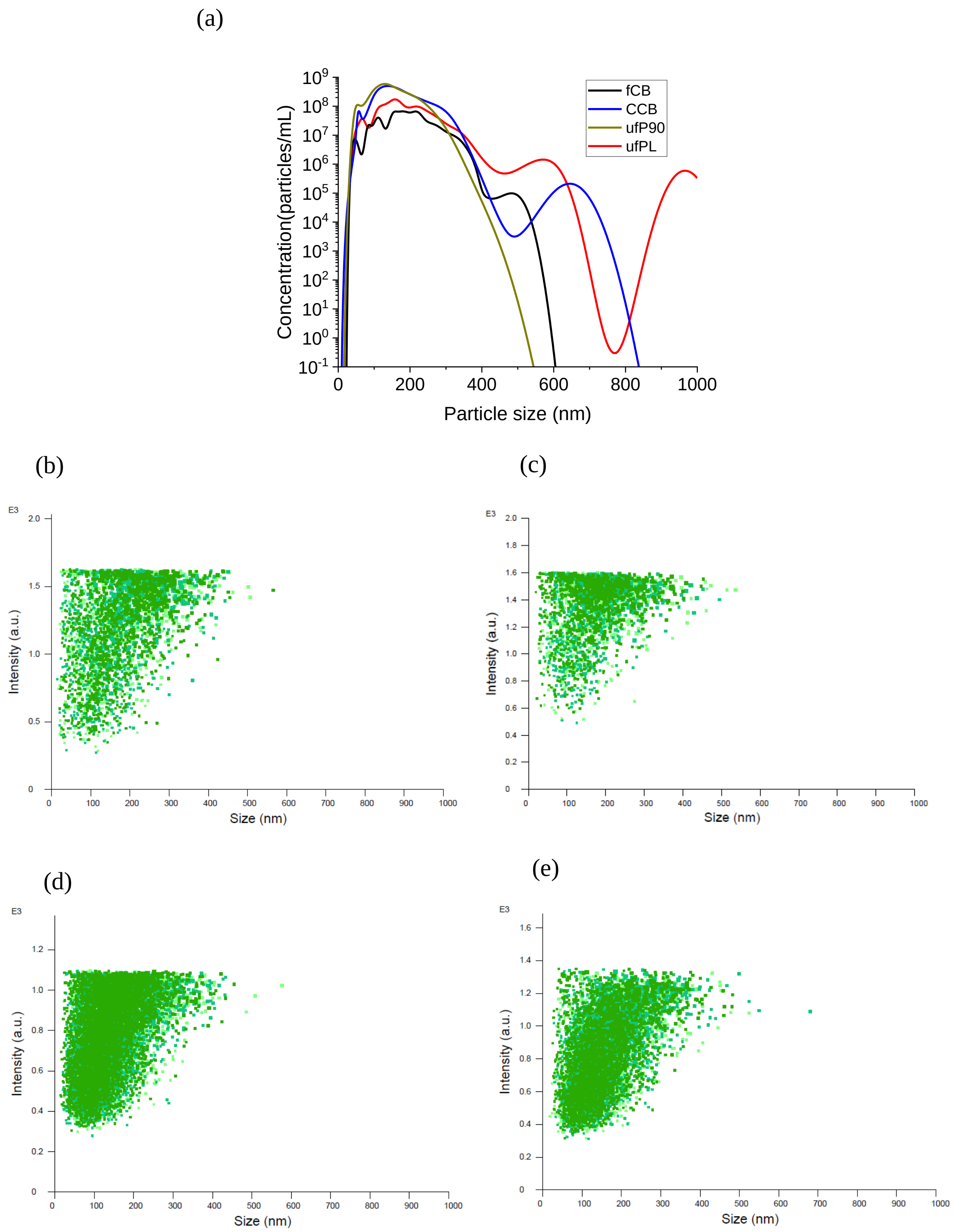

Figure S9: Sizing of CDCPs using Nanoparticle Tracking Analysis (NTA). (a) Size distribution of 4 different CDCPs employed in this study. (b-e) Size vs intensity from fCB, CCB, ufP90 and ufPL, respectively.

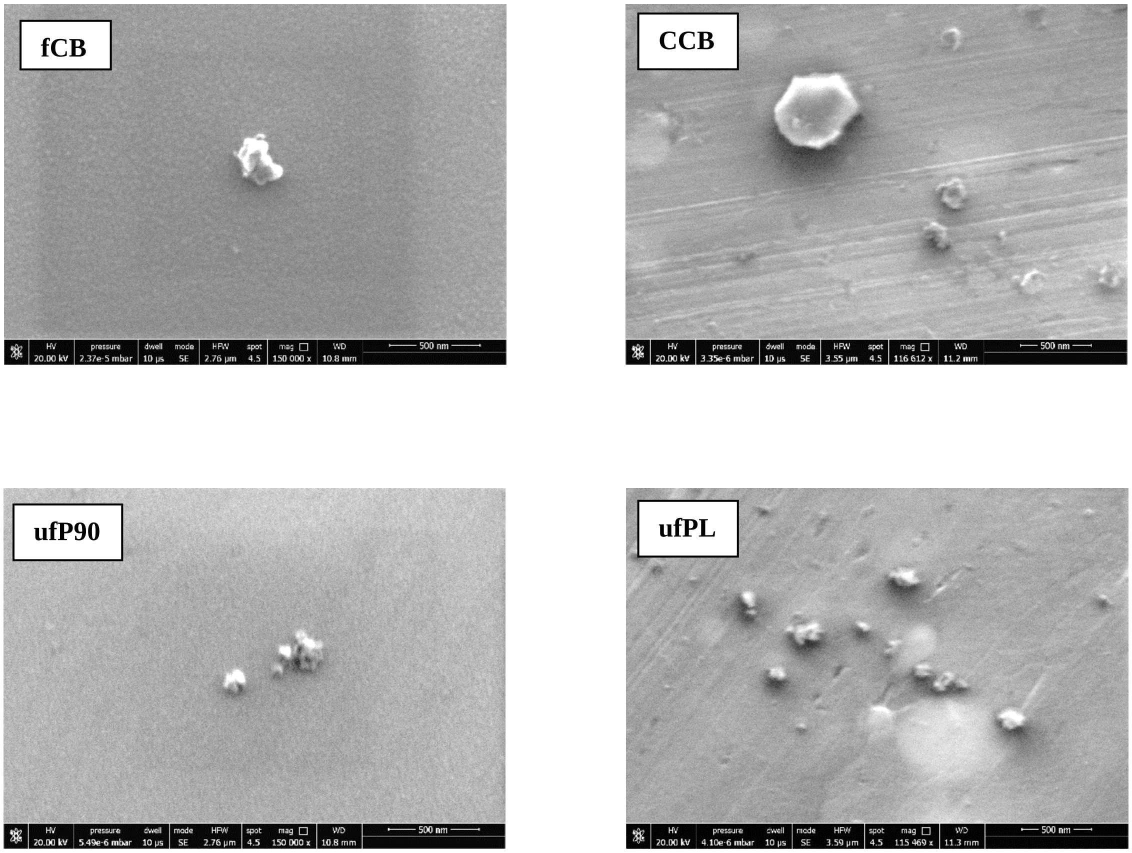

Figure S10: Scanning electron microscopy (SEM) images of CDCPs. Scale bar: 500 nm

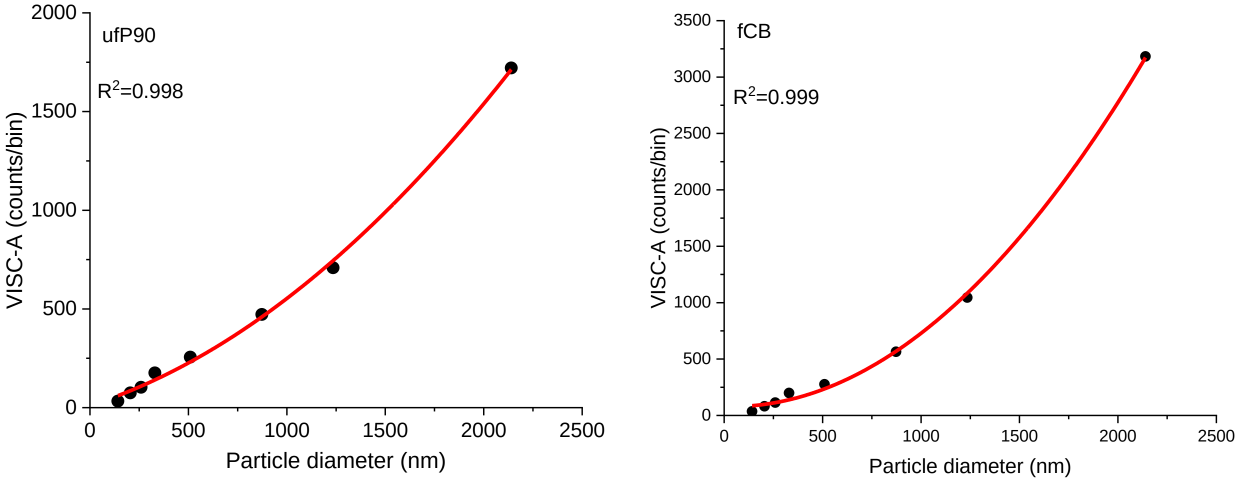

Figure S11: For CDCP sizes above 1 µm, the relationship between CDCP size and WL emission intensity becomes non-linear.

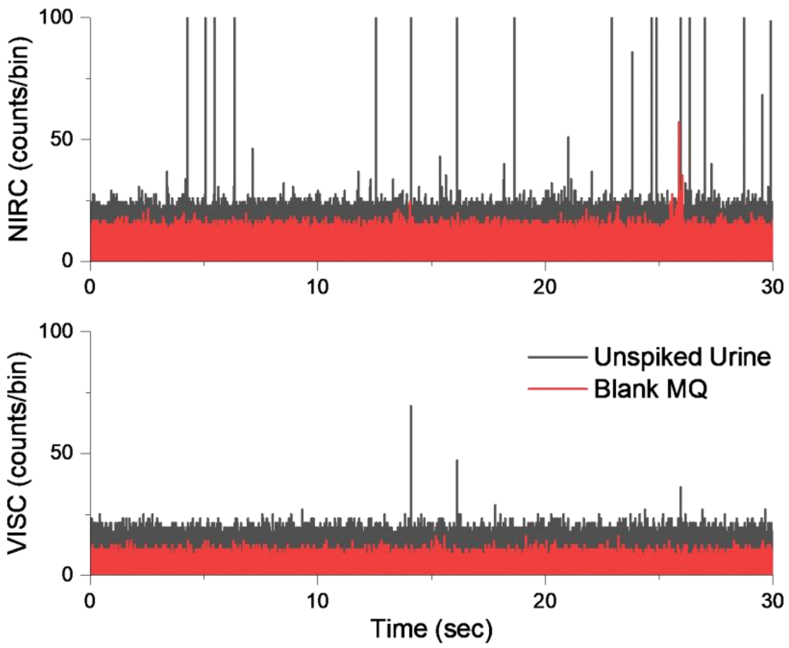

Figure S12: VISC and NIRC show a higher background signal from unspiked urine samples as compared to blank MQ. This elevated background from unspiked urine could be due to the intrinsic fluorescent and scattering species in urine samples.

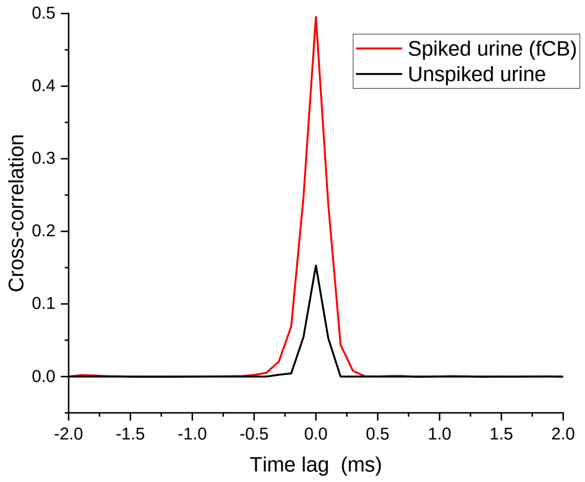

Figure S13: Cross-correlation between VISC and NIRC shows peaks at t=0 time lag for spiked urine and unspiked urine samples.

Calculations for actual count rate of APDs

After arrival of each photon pulse, an APD stays unresponsive for a specific amount of time which is called APD dead time. APD deadtime can underestimate the number of detected photons at very high photon count rates. Therefore at high photon counts, the count rate needs to be adjusted based on the theoretical calculations from the manufacturer's data.[1] Based on the dead time of the APDs, a linearity correction factor for each APD is provided by the manufacturer. The linearity correction factors and photon detection efficiency of APDs (at specific wavelengths) can be used to calculate the actual photon count rates for each APD. The typical deadtime for SPCM-AQRH-10 is 24 ns and the dark counts are 1500 cps as specified by the manufacturer's data. The photon detection efficiency is around 55% at 550 nm for VISC APD and around 65% at 780 nm for NIRC APD. The maximum photon count rate for VISC APD is 34 Mc/s while for NIRC APD is 38 Mc/s as specified by the manufacturer. Hence, the maximum photon count rate of 170 MHz for VISC and 190 MHz for NIRC can be achieved, respectively. From measurements on a mixture of 500 nm and 800 nm NPs, we could demonstrate that detectors can cover a large dynamic range without saturating. For both APDs, a 5th order polynomial fitting was used for the correlation of the linearity correction factor with the detected photon counts. The coefficients from the fitted data were used to estimate actual photon counts. This data was used for further photon burst identification for VISC and NIRC for our measurements. At the low count rates, the correction factor is almost equal to 1 as shown in **Figure S14**, however with an increase in the observed count rates the correction factor can go as high as 6.

$$Actual\ Count\ Rate_{Photons} = \frac{(OMCR \times CF\ \ ) - DC}{PDE}$$

Whereas;

$$OMCR = Output\ Module\ Count\ Rate$$

$$CF = CorrectionFactor@\ the\ Module\ CountRate$$

$$DC = Dark\ Count\ Module$$

$$PDE = Photon\ Detection\ Efficiency\ Module$$

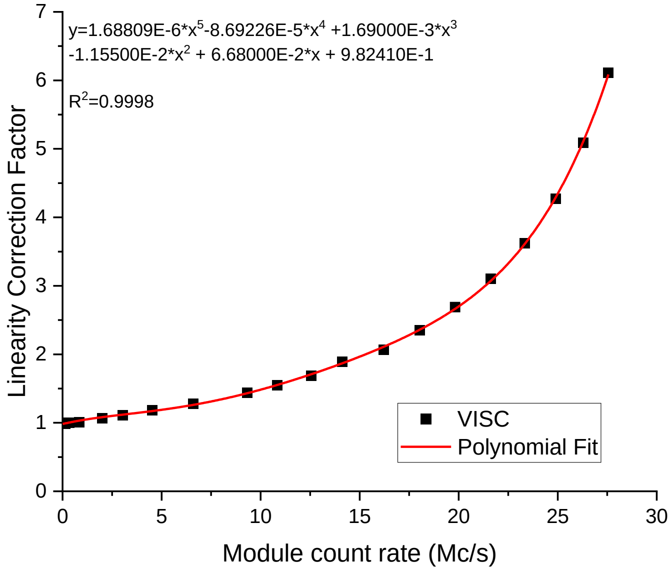

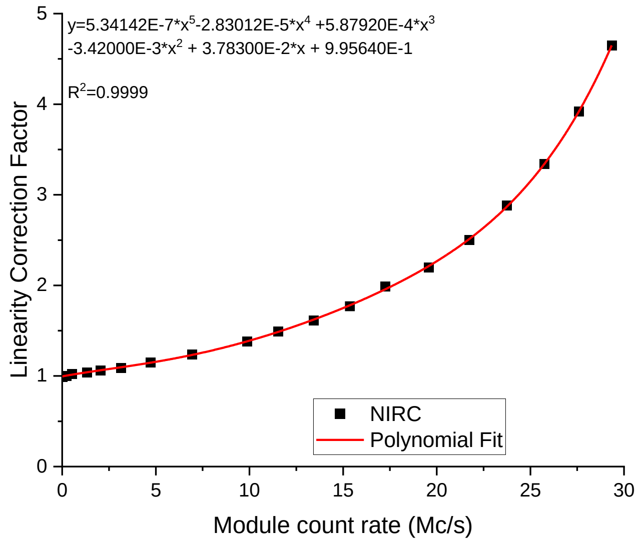

Figure S14: APD linearity correction factors for SPCM-AQRH-10 used for VISC and NIRC. These correction factors are based on the data provided by the manufacturer with deadtime of 24 ns and maximum count rates of ~34 Mc/s and ~38 Mc/s for VISC and NIRC APDs, respectively. A fifth-order polynomial fitting was used to determine each detector's response curve to determine correction coefficients. These correction factors were applied to the data obtained from both detectors individually.

Calculations for scattering cross-section and scattering intensity based on Mie Theory[2]:

The maximum projection angle of the objective with respect to the optical axis;

$$NA = n_m Sin(\alpha)$$

$$\alpha = Sin^{-1}\left(\frac{NA}{n_m}\right) \qquad Eq.(S1)$$

Where n_m is the refractive index of the medium.

The integration boundaries of the azimuthal angle (Φ_{min} and Φ_{max}) can be expressed as ;

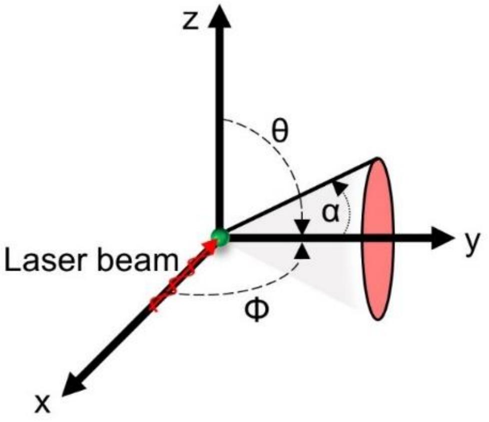

Figure S15: The polar coordinate system and the variables used to calculate the scattering intensity from a spherical particle illuminated using a fs-pulsed NIR laser polarized in xz plane. For side-scatter light detection using NIRC, the numerical aperture of the objective determines the acceptance angle α.

$$\Phi_{min} = Sin^{-1}\left(\frac{Sin(90° - \alpha)}{Sin(\theta)}\right) \qquad Eq.(S2)$$

$$\Phi_{max} = 180° - \Phi_{min} \qquad Eq.(S3)$$

Whereas θ is the polar angle with integration boundaries; $\theta_{min} = 90° - \alpha$ and $\theta_{max} = 90° + \alpha$.

The scattering cross-section (in nm^2) is given by[3];

$$\sigma_s = \int_{\Phi_{min}}^{\Phi_{max}} \int_{\theta_{min}}^{\theta_{max}} \frac{|S_1|^2 sin^2\Phi + |S_2|^2 cos^2\Phi}{k^2} sin\theta d\theta d\Phi \qquad Eq.(S4)$$

Whereas S_1 and S_2 are the amplitude scattering matrix elements and $k = \frac{2\pi n_m}{\lambda}$ is the wavenumber.

The values of the Mie scattering cross-section (in nm^2) for PS NPs can be calculated using the MieConScat software.[4]

Based on the scattering cross-section and the measured median scattering intensity from NIRC-A of PS NPs, we estimated a scaling factor through linear regression to relate measured median scattering intensity (*I*) from PS NPs to their theoretical cross-section σ_s in nm^2 ;

$$log_{10}(I) = log_{10}(C\sigma_s) \qquad Eq.(S5)$$

Where *C* is the scaling factor that relates the median measured scattering intensity (counts/bin) to the scattering cross-section (nm^2) of PS NPs. The same scaling factor can be used to estimate the scattering intensity of CDCPs of different sizes.

Although CDCPs might tend to aggregate in aqueous suspension, they are assumed sufficiently spherical in this study.[5]

	VISC OD filter	NIRC OD filter
200 nm	N/A	0.8
Mixture of 300,400, and 600 nm	N/A	1.5
Mixture of 500, and 800 nm	0.6	2
CDCPs suspensions	N/A	1.8

Table S1: Neutral density filters used with different optical densities (OD) for VISC and NIRC in case of different samples to avoid saturation of the detectors.

	Manufacturer's data<br>Average particle size (nm)	DLS hydrodynamic diameter (nm)<br>Ultrapure water
fCB	<500	361
CCB	150	456
ufP90	14	201
ufPL	13	228

Table S2: Average particle sizes based on manufacturer's data and hydrodynamic diameters measured in aqueous suspensions using DLS.

References:

(1) Sun, X.; Krainak, M. A.; Abshire, J. B.; Spinhirne, J. D.; Trottier, C.; Davies, M.; Dautet, H.; Allan, G. R.; Lukemire, A. T.; Vandiver, J. C. Space-Qualified Silicon Avalanche-Photodiode Single-Photon-Counting Modules. *J. Mod. Opt.* **2004**, *51–9* (10), 1333–1350. https://doi.org/10.1080/09500340408235276.

(2) de Rond, L.; Coumans, F. A. W.; Nieuwland, R.; van Leeuwen, T. G.; van der Pol, E. Deriving Extracellular Vesicle Size From Scatter Intensities Measured by Flow Cytometry. *Curr. Protoc. Cytom.* **2018**, *86* (1), 1–14. https://doi.org/10.1002/cpcy.43.

(3) Bohren, C. F. Absorption and Scattering of Light by Small Particles. *Absorpt. Scatt. Light by small Part.* **1983**. https://doi.org/10.1088/0031-9112/35/3/025.

(4) Rosenberg, P. D.; Dean, A. R.; Williams, P. I.; Dorsey, J. R.; Minikin, A.; Pickering, M. A.; Petzold, A. Particle Sizing Calibration with Refractive Index Correction for Light Scattering Optical Particle Counters and Impacts upon PCASP and CDP Data Collected during the Fennec Campaign. *Atmos. Meas. Tech.* **2012**, *5* (5), 1147–1163. https://doi.org/10.5194/amt-5-1147-2012.

(5) Wu, C.; Wu, D.; Zhen Yu, J. Quantifying Black Carbon Light Absorption Enhancement with a Novel Statistical Approach. *Atmos. Chem. Phys.* **2018**, *18* (1), 289–309. https://doi.org/10.5194/acp-18-289-2018.
